# Alcoholic EEG signal classification with Correlation Dimension based distance metrics approach and Modified Adaboost classification

**DOI:** 10.1016/j.heliyon.2020.e05689

**Published:** 2020-12-16

**Authors:** Sunil Kumar Prabhakar, Harikumar Rajaguru

**Affiliations:** a Department of Brain and Cognitive Engineering, Korea University, Anam-dong, Seongbuk-gu, Seoul 02841, South Korea; b Department of ECE, Bannari Amman Institute of Technology, Sathyamangalam, 638402, India

**Keywords:** Computer science, EEG, Alcoholism, Correlation distance, Distance metrics, Classification

## Abstract

The basic function of the brain is severely affected by alcoholism. For the easy depiction and assessment of the mental condition of a human brain, Electroencephalography (EEG) signals are highly useful as it can record and measure the electrical activities of the brain much to the satisfaction of doctors and researchers. Utilizing the standard conventional techniques is quite hectic to derive the useful information as these signals are highly non-linear and non-stationary in nature. While recording the EEG signals, the activities of the neurons are recorded from various scalp regions which has varied characteristics and has a very low magnitude. Therefore, human interpretation of such signals is very difficult and consumes a lot of time. Hence, with the advent of Computer Aided Diagnosis (CAD) Techniques, identifying the normal versus alcoholic EEG signals has been of great utility in the medical field. In this work, we perform the initial clustering of the alcoholic EEG signals by means of using Correlation Dimension (CD) for easy feature extraction and then the suitable features are selected in it by means of employing various distance metrics like correlation distance, city block distance, cosine distance and chebyshev distance. Proceeding in such a methodology aids and assures that a good discrimination could be achieved between normal and alcoholic EEG signals using non-linear features. Finally, classification is then carried out with the suitable classifiers chosen such as Adaboost.RT classifier, the proposed Modified Adaboost.RT classifier by means of introducing Ridge and Lasso based soft thresholding technique, Random Forest with bootstrap resampling technique, Artificial Neural Networks (ANN) such as Radial Basis Functions (RBF) and Multi-Layer Perceptron (MLP), Support Vector Machine (SVM) with Linear, Polynomial and RBF Kernel, Naïve Bayesian Classifier (NBC), K-means classifier, and K Nearest Neighbor (KNN) Classifier and the results are analyzed. Results report a comparatively high classification accuracy of about 98.99% when correlation distance metrics are utilized with CD and the proposed Modified Adaboost.RT classifier using Ridge based soft thresholding technique.

## 1 Introduction

One of the most common kinds of mental abuse is by means of both acute and chronic alcoholism [1]. Depending on the specific pattern of drinking and volume of alcohol consumed, the seriousness of it can be easily assessed. Alcoholism is a state where patients are obsessed with too much drinking alcohol, knowing well that alcohol can lead to a lot of impairments in the human brain and body and yet they cannot resist their desire to drink alcohol [2]. Too much of alcohol consumption leads to serious behavioral and cognitive problems in the human body by especially affecting the peripheral and central nervous systems. The patient develops an incapability to remember new things after chronic alcoholic consumption thereby taking a toll on the memory aspect too [3]. A major reason of worldwide mortality rates every year is due to alcoholism and its related disorders. Alcohol-related cancers are also on the significant rise affecting vital organs like stomach, liver, kidneys etc [4]. Alcoholism is also partially responsible for causing stomach ulcers, liver cirrhosis, pancreatic and gall bladder problems too. Alcoholism even contributes to deaths caused by accident, homicide, suicide, depression etc [5]. The social and personal relationships at colleges, offices, social gatherings, meetings etc is fully spoilt by alcoholism. Factors due to environment, genes and psychology contribute a lot to alcoholism. Because of such problems, alcoholism has to be definitely addressed in prominence so that early detection of it through simple and non-invasive techniques can save precious human lives [6]. Using the standard techniques, it is difficult to assess the cases related to alcoholism. Diagnosis of alcoholic patients in clinics is done by assessing the responses of alcoholic patients by measuring factors such as quantity of drinking, feeling of guilt, urge to drink incessantly, responses to criticism etc. Due to the societal stigmatization and fear, patients fail to reveal the exact information and so the rate of positive screening of alcoholism in hospitals is quite less. Therefore, with the advent of EEG, the measurement of the brain signals of the alcoholic patients can be easily done in a non-invasive manner [7].

For evaluating the various mental disorders like screening and diagnosis, EEG signals are predominantly utilized. By nature, EEG signals are highly dynamic and non-linear in nature and so it is utilized in various tasks such as classification of pilot mental states [8], drowsiness level detection [9], epilepsy detection [10], autism disorder detection [11], Alzheimer's disease detection [12], stroke analysis [13], consciousness and unconsciousness analysis [14], motor imagery classification [15], sleep related disorders [16], schizophrenia related disorders [17] etc. A more precise work for alcoholic EEG signal classification was done by Acharya et al in [18], where the alcoholic EEG signals was split from normal EEG signals by using non-linear features and Higher Order Spectra (HOS) features. Depending on the maximal weight matching concept, the functional connectivity in alcoholic EEG signals was done and evaluated by Zhu et al [19]. The alcoholic EEG signals were identified by their respective rhythms in [20]. The alcoholism disorder was explored by an EEG-based Machine Learning technique that utilizes resting state EEG features to classify the alcoholic patients by Mumtaz et al [21]. A wavelet filter bank technique which is orthogonal in nature and having three bands was utilized with Least Squares Support Vector Machine (LS-SVM) for identifying the alcoholic EEG signals by Sharma et al [22].

A medical expert who is trained to find out the fine variation in EEG signals between an alcoholic subject and normal subject for the diagnosis of alcoholism is quite difficult because the inspection of these subtle variations is difficult to analyze with the naked eye [23]. Moreover, to interpret the EEG frequency spectrum in a clinical manner, it involves the analysis of EEG frequency spectrum which is hectic to do. Owing to its strenuous and time-consuming nature, CAD is incorporated which involves both signal processing and soft computing techniques as a base to diagnose the disease using EEG signals [24]. A variety of signal processing techniques utilized for feature extraction are reported in [25]. Some of the commonly used soft computing techniques for analyzing the EEG signals are reported in [26]. To trace the deviation from the normal ones, certain linear and non-linear techniques [27] are utilized such as Hurst exponent, Lyapunov exponent, Fractal dimension etc.

In this paper, for the alcoholic EEG signals, initially the clusters are computed with the help of CD so that the features are extracted completely. Then the appropriate features are selected using distance metrics utilized here such as correlation distance, city block distance, cosine distance and chebyshev distance. Finally, it is classified with twelve suitable post classifiers for analyzing the alcoholic risk levels in EEG signals. The organization of the paper is as follows; In section 2, the materials and methods are discussed followed by the classification methodology utilized in section 3. Section 4 discusses the results and discussion and the paper is concluded in section 5 followed by suitable reference materials.

## 2 Materials and methods

Under this topic, the information about the dataset utilized, clustering through CD methodology for feature extraction and feature selection through distance metrics is explained.

### 2.1 Dataset utilized

**Figure 1:**
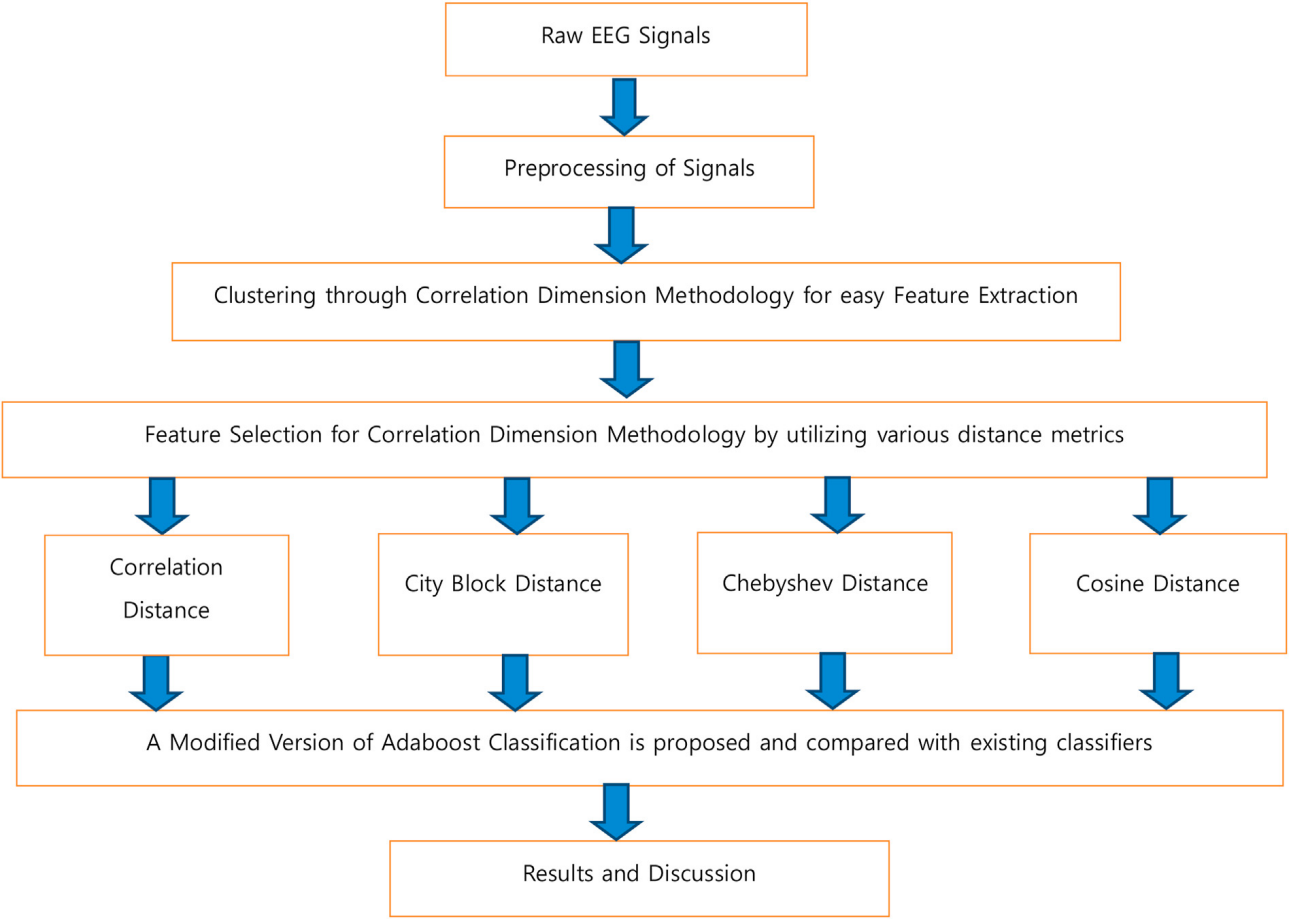
A simplified block diagram of the work for easy understanding.

The alcoholic EEG data utilized in this work was obtained from University of California, Irvine Knowledge Discovery database (UCI KDD), commonly known as UCI KDD archive [28]. To analyze the correlation between the EEG signals and alcoholic EEG signals, this dataset was acquired. Based on the standard 10/20 International montage, this dataset has the EEG recordings of totally 122 normal and alcoholic EEG patients. Based on the standard nomenclature of the American EEG committee, the electrode positions were placed in a standard manner. The impedance of the electrode was very much less than 5kΩ. Every subject has undergone 120 trials for various stimuli. Event- related potentials (ERP) were present in the EEG signals obtained by the 64 channel electrodes. The sampling rate of the system was about 256 Hz and a resolution range of 12 bits. Undesirable artifacts caused due to muscle movement, random eye movement and body movement were eliminated with a help of a simple preprocessing Independent Component Analysis (ICA) technique. With an artifact-free EEG signal, a reasonably accurate classification of alcohol levels is quite possible. But with artifacts, this problem enhances thereby leading to cases of false detection which in turn spoils the efficacy of the classification system and so it is very vital to perform pre-processing techniques initially. Then for both the normal and alcoholic EEG datasets, recordings with the appropriate data files were considered and stored in separate file formats each having a length of 2560 samples. The simplified version of the block diagram of the work to provide an easy understanding is given in Figure 1. The EEG signals are initially preprocessed and then clustered through CD methodology so that the features are extracted and finally efficient features are selected using various distance metrics. Then it is classified using suitable post classifiers for analyzing the alcohol levels from EEG signals.

### 2.2 Clustering through Correlation Dimension method for easy feature extraction

For physiological signals, the CD has always been a useful parameter and complements spectral analysis to a great extent [29]. Application of CD to analyze the EEG signals obtained from Parkinson's disease, Alzheimer's disease, glaucoma, and schizophrenia are widely reported in literature [30]. The CD acts as a good diagnostic parameter for the neural changes occurring in various neurological disorders. To explain the energy levels of various neural tasks also, this CD has been used always. For a set of random points, CDwis nothing but a simple measure of the dimensionality of the space occupied. To determine the fractal object dimension, the CD is of great use. Other techniques of measuring dimension are using techniques such as box-counting dimension, Hausdroff dimension, information dimension etc. Being straightforward and relatively easy to calculate, CD is widely used as it is less noisy with limited number of available points. For a particular set of Q points in a p- dimensional space, the feature representation f is expressed as:

(1) $$\vec{f}\left(j\right)=\left[f_{1}\left(j\right),f_{2}\left(j\right),....,f_{p}\left(j\right)\right],\;j=1,2,...,Q$$

Then the correlation integral C(ε)is computed as

(2) $$C\left(\varepsilon \right)=\lim_{Q\to \infty }\frac{h}{Q^{2}}$$

where h denotes the total number of pairs of points. As the total number of points leads to infinity, the distance in between them leads to zero and therefore the correlation integral will assume the form as follows

(3) $$C\left(\varepsilon \right)∼\varepsilon^{w}$$

A log-log graph of the correlation integral versus εgives an estimate of the CDw, if the points are pretty large and evenly distributed. For high dimensional objects, there are several ways for points to be much closer to each other. Therefore, the number of pairs closer to each other will be rising more for high dimension. Thus, in order to differentiate random and chaotic behavior, this technique is widely used. Therefore, with the help of this CD technique, clustering is done as follows.

**Figure 2:**
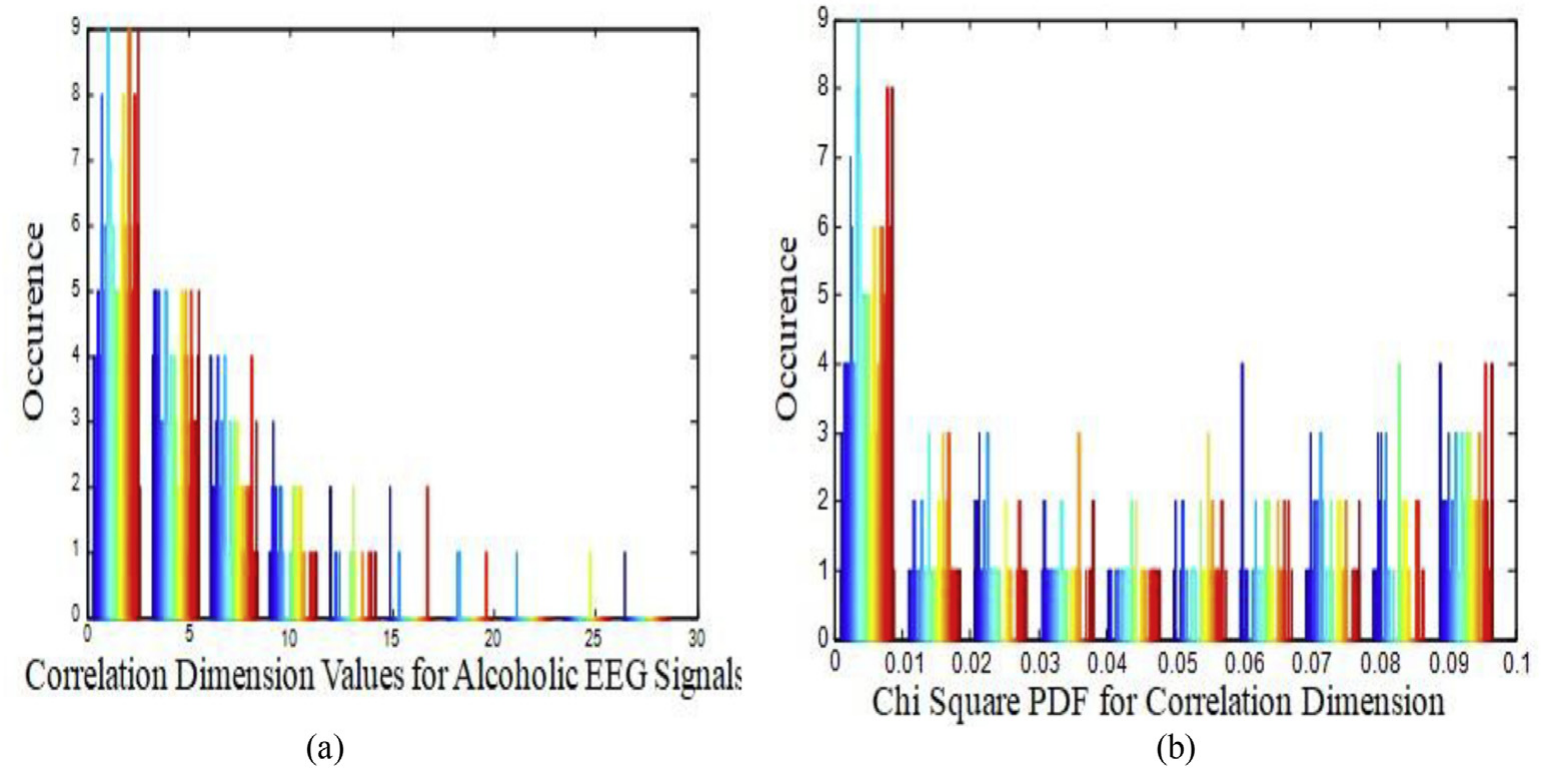
Histogram plot (a) CD for alcoholic EEG Signals for a patient.(b) Chi-Square PDF for C D with ten degree of Freedom for. alcoholic EEG Signal of a patient.

**Figure 3:**
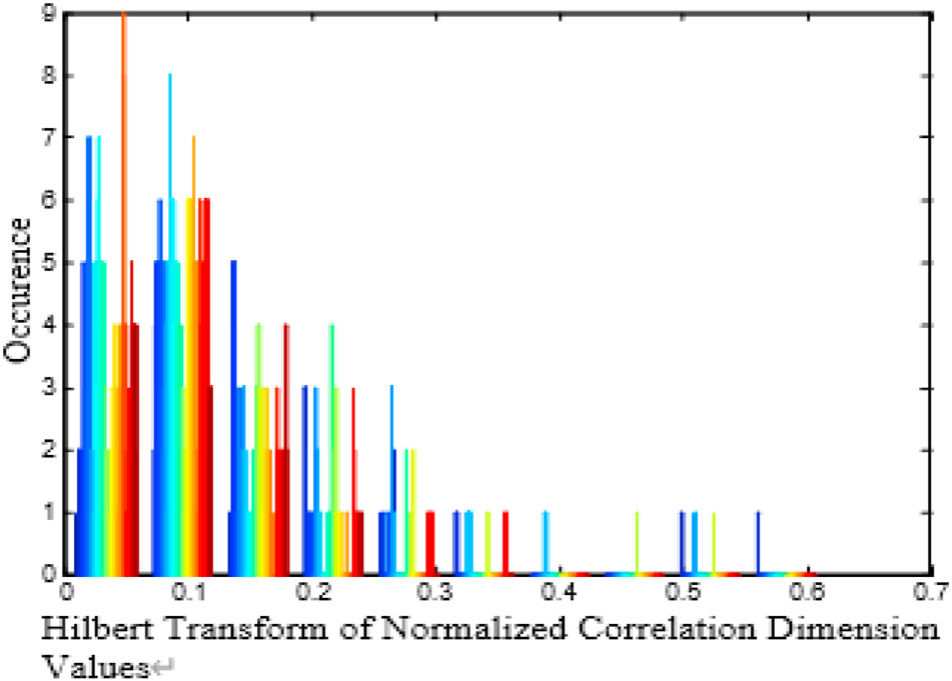
Histogram of Hilbert transform of normalized CD values for alcoholic EEG signal.

There are 2560 samples per channel and here there are 64 such channels available thereby making the total number of EEG samples as 163840. CD is utilized to reduce the 2560 samples per channel into 256 Correlated Dimension values. This accounts for ten times reduction in the size of the sample per channel. Hence, 16384 CD features are representing the EEG signal of an alcoholic patient. In order to identify the preservation of the non-linearity in the CD initially, we have to test the CD values with histogram plot. Histogram is a simple representation of repeated patterns based on their frequency of occurrence. It is observed from the analysis of CD values of EEG signals that non-linear dynamics is responsible for the presence of non- linearity in the histogram. The histogram for a patient is depicted in Figure 2(a). Table 1 shows the average value of statistical parameters like mean, variance, skewness, and kurtosis of CD features for alcoholic EEG signals of a subject. These CD values closely resemble the CD values reported by Acharya et al [18]. Figure 2(b) illustrates the Histogram of Chi Square Probability Density Function (PDF) for CD with ten degree of freedom for alcoholic EEG Signal of a patient. It is observed from Figure 2(b) that the histogram of Chi Square PDF is highly non-linear. Therefore, to explore further presence of non-linearity in the CD values, we subject the CD through Hilbert transform process and the histogram was plotted as shown in Figure 3.

**Table 1:** Average value of Statistical Parameters of CD Features for Alcoholic EEG Signal of a Subject.

Sl. No	Statistical Parameter	Numerical Value
1	Mean	4.091731
2	Variance	10.73556
3	Skewness	0.849836
4	Kurtosis	0.477126

As shown in Figures 2(a) and 3, the histogram pattern looks alike and hence, the presence of nonlinearity in the CD values are predominantly evident.

**Figure 4:**
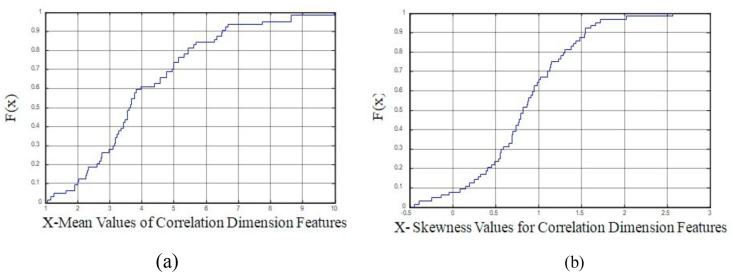
Cumulative Distribution Function (CDF) Plot for (a) CD Mean values b) CD Skewness values.

Figure 4(a), (b) depicts the Cumulative Distribution Function (CDF) plot for CD values for mean and skewness parameters. The presence of peak and valley points in Figure 4(a), (b) specifies the further evidence of non-linearity in the CD features.

**Figure 5:**
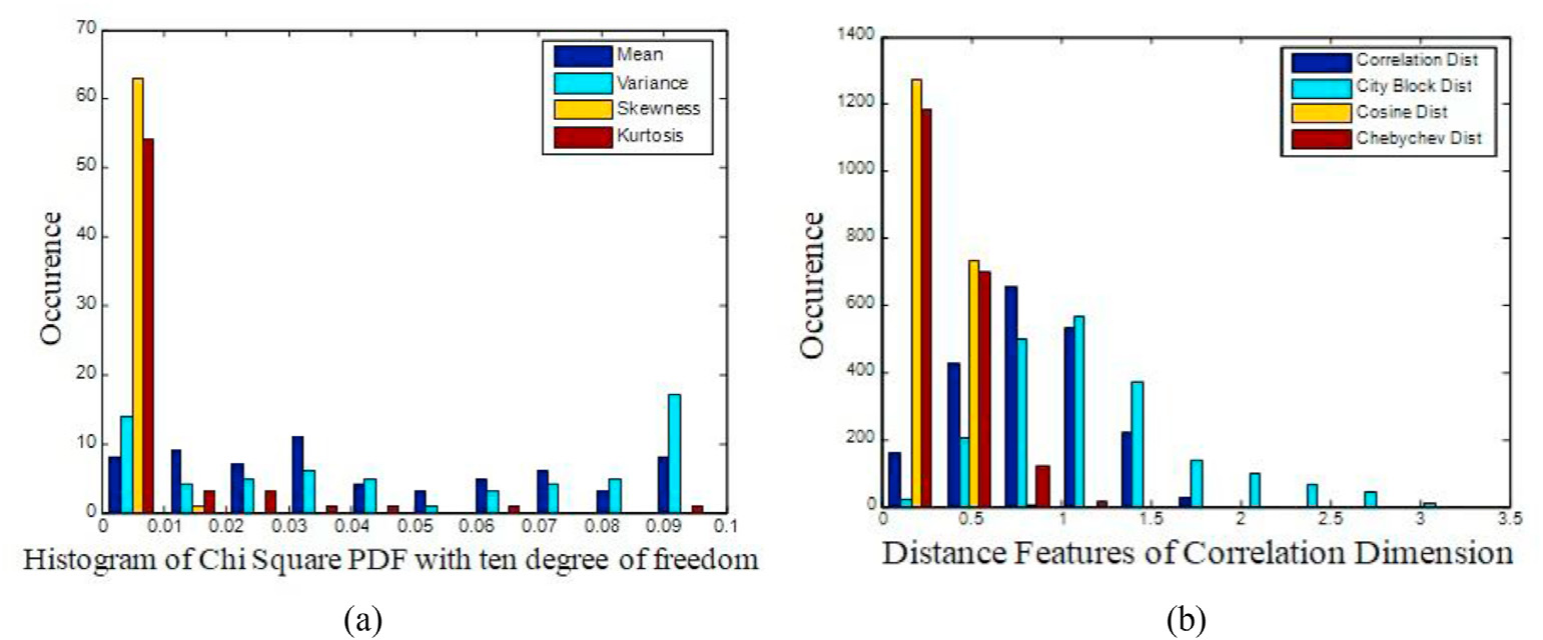
(a) Histogram of Chi Square PDF with ten degree of freedom for statistical parameters of CD (b) Histogram of CD with Distance features.

Figure 5(a) exhibits the histogram of Chi Square PDF with ten degree of freedom for statistical parameters of CD values. The peaked distribution of skewness and kurtosis in Figure 5(a) indicates the non-linearity in the CD values. The mean and variance exhibit the highly flatten condition of the histogram which shows the abnormal trend in the CD values. Figure 5(b) shows the Histogram of CD with four selected Distance features. Figure 5(b) illustrate that the reduced distance feature selection process will ease the burden of the classifiers.

### 2.3 Feature selection through distance metrics

Once the clustering is done through CD methodology, then the efficient features are selected from the clusters through distance metrics concept. The distance metrics analyzed here for feature selection are Correlation Distance, City Block distance, Cosine distance and Chebyshev distance measures.

A. Correlation Distance

The correlation distance is a simple measure of dependence between 2 paired random vectors of equal dimension [31]. If the random vectors are independent, then the correlation distance coefficient is considered zero. Between two random vectors or variables, the linear and non-linear association is easily measured by Correlation Distance. Pearson's correlation can easily detect the linear relation between two random variables whereas Correlation distance can detect the linear and non-linear relation between two random variables.

Let $\left(M_{k},P_{k}\right)k=1,2,...,p$ be a statistical sample considered from a pair of real valued random variables(M,P). The correlation distance of two random variables is obtained by finding the ratio of their covariance distance by the product of their standard deviation distance. The correlation distance is mathematically expressed as

(4) $$dCor\left(M,P\right)=\frac{dCov\left(M,P\right)}{\sqrt{dVar\left(M\right)dVar\left(P\right)}}$$

The correlation distance has three important properties such as:

i) $0\leq dCor_{n}\left(M,P\right)\leq 1$ and 0≤dCor(M,P)≤1, this is different when compared to Pearson's correlation as it can be negative
ii) dCor(M,P)=0, if and only if MandPare independent
iii) $dCor_{n}\left(M,P\right)=1$denotes that the linear subspaces designated by Mand Psamples are almost equal.
B. City Block Distance

It comes under taxicab geometry. It is a type in which the general metric of Euclidean geometry is replaced by a new metric. In such a case, the distance between the two points is the sum of the absolute differences of their respective coordinates. It is also called as Manhattan, rectilinear and taxicab metric [32]. It is defined as the sum of the lengths of the projections of a particular line segment between the points onto the coordinate axis. It is represented as follows:

(5) $$d=\sum_{i=1}^{n}\left|m_{i}-p_{j}\right|$$

C. Chebyshev Distance

The Chebyshev distance between 2 vector points mandp, with standard coordinates $m_{i}$and $p_{i}$respectively is expressed as

(6) $$D_{Chebyshev}\left(m,p\right)=\max_{i}\left(\left|m_{i}-p_{i}\right|\right)$$

It is also called as chessboard distance as in the game of chess, the minimum number of moves required by a king to go from one square to another equals to the Chebyshev distance between the centers of the square [33].

D. Cosine Distance

Cosine similarity is a method utilized to measure how similar the entities are irrespective of their size [34] and is expressed as

(7) $$\begin{array}{ll} \mathrm{Cosine} & Similarity \end{array}=\frac{\sum_{i=1}^{n}m_{i}p_{i}}{\sqrt{\sum_{i=1}^{n}m_{i}^{2}}\sqrt{\sum_{i=1}^{n}p_{i}^{2}}}$$

Therefore, Cosine Distance = 1 – Cosine Similarity.

**Figure 6:**
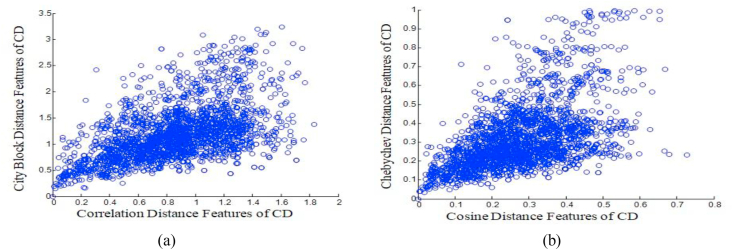
Scatter Plot (a) between Correlation and City block Distance Features of CD Values (b) between Cosine and Chebyshev Distance Features of CD Values.

The scatter plot depicts how the features are closely correlated to each other. As from Figure 6(a), it is identified that a scatter plot of correlation and City block distance features are closely correlated one. From this plot the target value is selected as the maximal regression point which is identified as 0.45. Selection of target value for the classifiers will be easy in the plot due to the center cluster of the plot is settled at 0.45. Therefore, the target for the classifiers is set at 0.45.

## 3 Classification methodology

The obtained best features through distance metrics are then classified with the help of the standard Adaboost.RT algorithm, modified version of Adaboost.RT algorithm, Random Forest classifier, K means Classifier, ANN like RBF and MLP, SVM with Linear, Polynomial and RBF kernels, NBC and KNN classifier.

### 3.1 The Standard Adaboost.RT algorithm

The Adaboost.RT algorithm was proposed by Solomatine & Sherstha [35]. For problems dealing with regression, it is a good boost algorithm where R represents regression and T represents threshold respectively. The Adaboost.RT algorithm in its original form is explained in Algorithm 1.

Algorithm 1 Standard Adaboost.RT algorithm.

The following are fed as input:

(i) a specific sequence of ‘*q’* samples $\left(a_{1},b_{1}\right),...,\left(a_{q},b_{q}\right)$where output b∈R
(ii) weak learning procedural step
(iii) The number of iterations specified as integer Z
(iv) For approximating and differentiating the correct from incorrect predictions, a threshold ψis used where (0<ψ<1)

The following are initialized:

(i) Iteration z=1
(ii) Distribution Dz(j)=1q for all j
(iii) Error rate $\varepsilon_{z}=0$

The learning steps are as follows while iterating z≤Z

Step 1: The weak learning is called for and it is provided with approximate distribution $D_{z}$

Step 2: The regression model is then built up as $h_{z}\left(a\right)\to b$

Step 3: For each training example, the Absolute Relative Error (ARE) are calculated as

$$ARE_{z}\left(j\right)=\left|\frac{h_{z}\left(a_{j}\right)-b_{j}}{b_{j}}\right|$$

Step 4: The error rate of $h_{z}\left(a\right)$is calculated as $h_{z}\left(a\right):\varepsilon_{z}=\sum_{j:ARE_{j}\left(j\right)>\psi }D_{z}\left(j\right)$

Step 5: The $\beta_{z}=\varepsilon_{z}^{c}$, where the power coefficient is denoted as c

Step 6: The distribution $D_{z}$is updated as follows:

If $ARE_{z}\left(j\right)\leq \psi$, then $D_{z+1}\left(j\right)=(D_{z}\left(j\right)/Y_{z})\times \beta_{z}$, else $D_{z+1}\left(j\right)=D_{z}\left(j\right)/Y_{z}$, where $Y_{z}$denotes a normalization factor chosen such that $D_{z+1}$ is its respective distribution.

Step 7: Finally, set z=z+1

The final output hypothesis is as follows:

$$h_{hin}\left(a\right)=\frac{\Sigma_{z}\left\{\left(\log\left(1/\beta_{z}\right)\times h_{z}\left(a\right)\right)\right\}}{\Sigma_{z}\left(\log\left(1/\beta_{z}\right)\right)}$$

The regression problem is being predicted as the binary classification problem by the Adaboost.RT algorithm. Based on the Absolute Relative Error (ARE), this algorithm can predict whether the samples are correct or incorrect. If the ARE of a sample value is not large than the threshold ψ, then it is termed as correct predictor otherwise it is turned as incorrect predictor. In this classification problem, it gives an idea about correct classification and misclassification. To those weak learners, very large weights are being arranged by the algorithm in order to reach a high prediction rate. The final hypothesis is the simple combination of the weak learner outputs using the respective computed weights. Due to the requirement of manual selection of threshold ψ, Adaboost.RT has a slight problem and sometimes considered unstable. Sometimes it may cause overfitting and produces a low convergence efficiency.

### 3.2 Modified Adaboost.RT algorithm based on Ridge, Lasso and Soft Thresholding

The proposed modification is that of the Ridge, Lasso and Soft Thresholding based Adaboost.RT algorithm. Based on the intrinsic property of the input data samples, the initial threshold values can be easily determined by Algorithm 2. When the data suffers from multicollinearity, ridge regression technique is widely used [36]. Here the Least squares estimates are unbiased, but it has a very high variance value. Hence to the regression estimates, adding degree of bias reduces the standard errors. Therefore, the prediction errors are decomposed into two sub-components due to the bias and variance. Through the shrinkage parameter(λ), the multicollinearity problem is solved by Ridge regression. The equation is simple and expressed as $\underset{\beta \in {ℜ}^{p}}{\mathrm{argmin}}{\left\|b-A\beta \right\|}_{2}^{2}+\lambda {\left\|\beta \right\|}_{2}^{2}$, where it indicates the combination of the loss and penalty component. The first term denotes the least square term and the second term denotes the summation of the coefficient β. In order to reduce the parameter to a very low variance, this term is added. The operation of Lasso regression is quite similar to ridge regression and helps to penalize the absolute size of the regression co-efficients [37]. In order to improve the accuracy and to mitigate the variability, the model is used and is expressed as $\underset{\beta \in {ℜ}^{p}}{\mathrm{argmin}}{\left\|b-A\beta \right\|}_{2}^{2}+\lambda {\left\|\beta \right\|}_{1}$. Instead of squares, absolute values are used in the penalty function and that is a major difference in the lasso regression when compared to ridge regression. Therefore, it leads to penalizing certain parameter estimates to turn to negligible condition. If the penalty applied is larger, then the estimates tend to shrink towards zero.

Algorithm 2 Modified Adaboost.RT algorithm based on Ridge, Lasso and Soft Thresholding

(1) The following should be considered as input: sequence of q samples $\left(a_{1},b_{1}\right)....\left(a_{q},b_{q}\right)$, where output b∈R, weak learner and total number of iterations denoted as Z.
(2) The following are initialized: iteration index z=1, distribution Dz(j)=1qfor all j, the weight vector:$w_{j}^{z}=D_{z}\left(j\right)$for all j, error rate $\varepsilon_{z}=0$
(3) The following are iterated while z≤Z
  a) The weak learner is called $WL_{z}$, and it is provided with appropriate distribution represented as $p^{\left(z\right)}=\frac{w^{\left(z\right)}}{Y_{z}}$, where $Y_{z}$ is a normalization factor chosen and $p^{\left(z\right)}$represents the distribution.
  b) The regression model is built as $h_{z}\left(a\right)\to b$ and then ridge regression and lasso regression are applied to it.
  c) To introduce the soft thresholding concept now, the signal received is initially expressed as l[n]=m[n]+v[n], where m[n]denotes the unknown signal which has to be detected and v[n]represents the Gaussian distribution noise factor. The soft thresholding is implemented as: $\overset{⌢}{M}=\left\{ \begin{array}{ll} l-\mathrm{sgn}\left(l\right)T & if\left|l\right|\geq T\\ 0 & if\;\left|l\right|<T \end{array}\right.$
  d) Error is calculated as: $e_{z}\left(j\right)=h_{z}\left(a_{j}\right)-b_{j}$
  e) The error rate is calculated as $\varepsilon_{t}=\Sigma_{j\in p}p_{j}^{\left(z\right)}$, where $P=\left\{j|\left|e_{z}\left(j\right)-{\bar{e}}_{z}\right|>\lambda \sigma_{z}\right\},j\in \left[1,q\right]$, ${\bar{e}}_{z}$ denotes the expected value and $\lambda \sigma_{z}$is denoted as robust threshold value, where $\sigma_{z}$denotes the Standard deviation. Here the relative factor λ is told as λ∈(0,1).If $\varepsilon_{z}>1/2$, then prioritize Z=z−1and terminate the loop.
  f) Initialize $\beta_{z}=\varepsilon_{z}/\left(1-\varepsilon_{z}\right)$
  g) The contribution of $f_{z}\left(a\right)$ is calculated to the final result: $\alpha_{z}=-\log\left(\beta_{z}\right)$.
  h) The weight vectors are updated as:

If j∈P, then $w_{j}^{\left(z+1\right)}=w_{j}^{\left(z\right)}\beta_{t}$;

Else ${w_{j}}^{\left(z+1\right)}=w_{j}^{z}$

i) Finally set z=z+1

4) The $\alpha_{1},...,\alpha_{Z}$is normalized so that$\sum_{z=1}^{Z}\alpha_{z}=1$. The final hypotheses output is determined as: $h_{hin}\left(a\right)=\sum_{z=1}^{Z}\alpha_{z}h_{z}\left(a\right)$

For the approximate error distribution, the standard deviation is utilized as a criterion for every iteration of this proposed algorithm. If the data points are close to the mean values, then it indicates that there is a low Standard Deviation and on the other half, if the data points are far from the mean values, then it indicates that there is a high Standard Deviation. For a particular set of predictions, Standard deviation serves as a standard for uncertainty. From the predictions to the original values, if the average distance obtained is small, then the regression model utilized here need not be revised. The prediction accuracy set is generally low if the respective sample points falls outside the range of values. In this proposed method, the approximate error of the $z^{th}$weak learner, $WL_{z}$, for an input dataset could be indicated as one standard distribution with parameters $\mu_{z}\pm \lambda \sigma_{z}$, where $\mu_{z}$denotes the expected value and $\sigma_{z}$denotes the standard deviation and λdenotes the relative factor ranging from 0 to 1. The threshold value for $WL_{z}$ is assessed by the scaled standard deviation$\lambda \sigma_{z}$. The generation of small prediction errors is done by the trained weak learners so thatεz<12. For the regression errors, the obtained means are fluctuating around zero and is within a small range. The individual samples can fully determine the standard deviation$\sigma_{z}$. For most cases $\sigma_{z}$ is very large than most outputs and is located within the ranges $\left[-\sigma_{z},+\sigma_{z}\right]$, thereby contributing to the instability of the boosting process. The relative factor λis implemented to standard deviation to standardize the stability of the threshold value. If the samples are present within the threshold range $\left[-\lambda \sigma_{z},+\lambda \sigma_{z}\right]$, it is considered as accepted samples and if it falls outside the threshold ranges, then it is called rejected samples.

### 3.3 Random forest classifier with bootstrap resampling technique

The feature values obtained through distance metrics are utilized as input to a classifier with the sole intention of classifying the alcoholic signals. The RF classifiers mainly depend on the classification results of various classification trees [38]. Then a random vector is assigned to each tree and these assigned Random vectors has the same kind of distribution and are not dependent on one another. Therefore, to perform classification, both the training data and the random vector assigned provide the necessary support to tree to perform the classification. The validation of the classification performance is done using 10-fold cross validation technique. Then the classification performance parameters such as sensitivity, specificity and accuracy are used to evaluate the method.

The RF classification algorithm is utilized in two phases. Initially, using the bootstrap resampling technique the extraction of the subsamples from the original samples are done. Secondly, the classification of the decision trees is done and then a simple vote is implemented with the largest vote of classification obtained as the ultimate result of the prediction and this procedure is shown in Algorithm 3. For a training dataset $F=\left\{{\left(A_{j,}B_{j}\right)}_{j=1}^{N}|A_{j}\in {ℜ}^{M},B\in \left\{1,2,...,q\right\}\right\}$, where $A_{j}$are features, Brepresents a class response feature, Ndenotes the total number of training samples, Mdenotes the total number of features and a RF model told in Algorithm 3. Assume${\overset{⌢}{B}}^{h}$is the predictor of tree $T_{h}$ given inputA. The prediction output of random forest with Htree is given as follows

(8) $$\overset{⌢}{B}=\begin{array}{ll} majority & vote \end{array}{\left\{{\overset{⌢}{B}}^{h}\right\}}_{1}^{H}$$

Since every tree is obtained from a bagged sample set, it is grown with only two third of the sample in the training set and it is called as in-bag samples. To estimate the prediction error, out-of-bag (OOB) samples are utilized which are only about one third of the samples left out. The out of bag predicted values is expressed as ${\overset{⌢}{B}}^{OOB}=\left(1/\left\|\theta_{j}\right\|\right)\sum_{h\in {\theta_{j}}^{\prime }}{\overset{⌢}{B}}^{h}$, where $\theta_{j^{\prime }}=F/\theta_{j}$, where jrepresents the in bay sample index and $j^{\prime }$represent the out of bay sample index. $\left\|\theta_{j^{\prime }}\right\|$ represents the size of the OOB sub dataset. The prediction error of OOB is expressed as

$Err^{OOB}=\frac{1}{N_{OOB}}\sum_{j=1}^{N_{OOB}}\varepsilon \left(B,{\overset{⌢}{B}}^{OOB}\right)$where ε(•) denotes the error function and $N_{OOB}$represents the sample size of OOB.

Algorithm 3 Random Forest Classification with Bootstrap Resampling Technique

Input: $F=\left\{{\left(A_{j},B_{j}\right)}_{j=1}^{N}|A\in {ℜ}^{M},B\in \left\{1,2,....,q\right\}\right\}:$the training dataset

H: the number of trees,

msub: subspace size

Output: A random forest RF

(1) For h←1to Hdo
(2) A bagged subset of samples is drawn to $F_{h}$ from F
(3) While (failure of stopping criteria) do
(4) Random selection of m sub features are done
(5) For m←1 to ‖msub‖do
(6) Reduction in the node input is computed
(7) The feature which mitigates the inputs to the utmost level is chosen and then the node is divided into 2 children nodes.
(8) The Htrees are combined to form a random forest.

### 3.4 ANN based classifier

To process neurobiological signals extracted from EEG, ANN's are widely used [39]. In this paper, two kinds of ANNs are analyzed to determine the most convenient configuration.

i) RBF network with 64 neurons in the hidden layer, 32 input and 1 output linear neurons.
ii) MLP, where 32 input linear neurons and one output neuron with logistic activation function is present. It has a single hidden layer comprising 64 neurons with hyperbolic tangent as an activation function. Recognizing the non-linear data requires more hidden layers with non-linear activation functions and that is provided in MLP

The training MSE and testing MSE for both the RBF architecture and MLP architecture are shown in Table 2 and Table 3 respectively. In this work, only 32-64-1 architecture has been utilized for both RBF and MLP as it produced a very less training and testing MSE.

**Table 2:** MSE analysis for RBF architecture.

Sl.No	RBF Architecture	Training MSE	Testing MSE
1	32-1-1	0.00019321	0.002506079
2	32-2-1	0.000545623	7.84E-06
3	32-4-1	0.000001	8.56625E-06
4	32-8-1	9.39067E-05	7.05094E-06
5	32-16-1	1.156E-05	2.84625E-06
6	32-32-1	1.96E-06	4.84E-06
**7**	32-64-1	1.44E-06	4.69844E-07
8	32-128-1	1.76241E-05	1.96E-06
9	32-148-1	2.90631E-05	4.41E-06

**Table 3:** MSE analysis for MLP architecture.

Sl.No	MLP Architecture	Training MSE	Testing MSE
1	32-1-1	0.000163929	0.000001
2	32-2-1	8.836E-05	7.84E-06
3	32-4-1	1.369E-05	4E-06
4	32-8-1	0.000036	6.4E-06
5	32-16-1	4.80644E-05	5.24E-06
6	32-32-1	1.31567E-05	1.156E-05
**7**	32-64-1	8.1E-07	1E-08
8	32-128-1	4.84E-06	4.84E-06
9	32-148-1	1E-06	0

### 3.5 Support Vector Machine

Here SVM classifier with linear kernel, polynomial kernel order 1 and RBF kernels are utilized [40]. A hyperplane is searched as a decision surface in SVM which tends to split the two classes to the maximum extent by a very high margin. The hyperplane which is separated will be inclined to the shortest line in a perpendicular aspect thereby separating the convex hulls of each class. Assuming, a.v+c=0is the separating hyperplane, where v is its normal. For $\left\{a_{i},z_{i}\right\},a_{i}\in {ℜ}^{n},z_{i}=\left\{-1,1\right\},i=1,2,...,N$separable data, the wisely chosen optimum boundary with maximum margin criterion is obtained by mitigatingF=‖v‖, provided $\left(a_{i}.v+c\right)z_{i}\geq 1$, for all i. The support vectors R∈{1,2,....,N}subset of the training data are the solutions obtained for optimum boundary level $v_{0}=\Sigma_{i}\alpha_{i}z_{i}a_{i}$and is attained in dual form easily. In order to get the decision boundary in an optimum manner, Quadratic programming techniques are utilized to solve this optimization problem where the random vectors are linearly combined [38]. With the aid of polynomial and RBF kernels, the non-linear boundary problems can be easily obtained. As far as the hyper parameter selection is done for SVM-RBF Classifier, the gamma value of the RBF kernel is selected such as starting from 0.2, 0.4, 0.6 and up to 2.6. While experimenting it is observed that for Gamma value of 2.0, lower MSE of 4.00E-08 is attained at the number of iterations of 250. Furthermore, in this case an increase in the number of iteration is depicted as reduced MSE value. For all other gamma values, the SVM-RBF classifiers is either plugged with local minima or has a flattened MSE effect. Therefore, for SVM-RBF Classifier the Gamma value is selected as 2.0. For SVM-Polynomial method, the order of the polynomial is controlled by using grid search method. For SVM-linear method, random search procedure is used to select the hyper parameter.

### 3.6 Naïve Bayesian Classifier

One of the most famous to predict the specific probability of a class membership is Naïve Bayesian classifier [41]. In bio signal processing, a high accuracy with a very low computation time is yielded by Naïve Bayesian process. The Naïve Bayesian depends on an assumption that the particular influence of an observation in a specific class is independent of the values present or obtained from other observation. Therefore, a class conditional independence is always assumed because of this criterion. Let $Z=\left\{z_{1},z_{2},...,z_{q}\right\}$is a particular sample that consists of ‘*q’* points. Assume ‘*P*’ is a hypothesis for each set of Zthat belongs to a particular class. In Bayes rule, Zis considered as an evidence and finds to match each point of Zis considered as an evidence and finds to match each point of Zto the probability class containing the highest posteriori.

### 3.7 K means classifier

It is used to solve a clustering problem and it comes under the category of unsupervised classification technique. For a given population, this technique separates it into a number of clusters on the position of K centroids for every cluster [42]. By mitigating the Euclidean distance between the cluster centroid and the observation point, it can be easily achieved. Every observation which pertains to the given population is related to the nearest centroid. To obtain the initial clustering level, this step is repeated at every iteration and therefore the calculation of the new K centroids is done.

### 3.8 K-nearest neighbor classifier

As it is quite robust with huge noisy datasets, it is one of the most commonly used classification techniques. It is also adaptive in nature and is known for the prediction of unknown data. Based on the frequency of a class from its nearest neighbours present in a particular feature space, the classification task is performed in such an easy manner. To define the distance in the KNN algorithm, various distance metrics are utilized. Depending on the training session, the nature of distance utilized here in this work is Euclidean [43]. The similarities with K-nearest neighbours are calculated and the class which corresponds to the maximum number of votes is assigned as the output class of the instance. As far as the hyper parameter selection is done for KNN Classifier, the value is set as 4 initially and for various values such as K = 4,6,8, the procedure is carried out to trace the optimum number of iterations for a low MSE value and after experimentation K = 4 gave a lowest MSE.

## 4 Results and discussion

In this section, the performance metrics analysis, and the summary of the previous works with the obtained results are compared and analyzed.

### 4.1 Performance metrics analysis

By utilizing the clustering technique through means of CD method and then utilizing distance metrics as suitable feature extraction technique, it is finally classified with the help of post classifiers. For the risk level classification of alcohol clustering and distance metrics feature extraction from EEG signals, based on the parameters like Sensitivity, Specificity, Performance Index and Accuracy, the average results are computed. The Mean Square Error (MSE), Good Detection Rate (GDR) and Error Rate analysis for the classifiers is also computed. In this work, k fold cross validation is used. Initially the dataset comprising the features is split into ‘*k*’ equal size points. k−1groups are then utilized to check the performance in every step. The validation is repeated for knumber of times and here k=10. Therefore, 90% of the data was utilized for training and 10% for testing. The computation of the average if all performance metrics at the end of 10-fold process is done.

The mathematical formulae for the Performance Index (PI), Sensitivity, Specificity and Accuracy are expressed as follows:

(9) $$PI=\left(\frac{PC-MC-FA}{PC}\right)\times 100$$

where Perfect Classification – PC, Missed Classification – MC and the False Alarm – FA. The Sensitivity, Specificity and Accuracy measures are mathematically defined by the following

(10) $$Sensitivity=\frac{PC}{PC+FA}\times 100$$

(11) $$Specificity=\frac{PC}{PC+MC}\times 100$$

(12) $$Accuracy=\frac{Sensitivity+Specificity}{2}$$

GDR: It is one of the most significant criteria of a detector, representing the ability of a detector in successful detection and specified as

(13) $$GDR=\left(\frac{\left[PC-MC\right]}{\left[PC+FA\right]}\right)\times 100$$

The MSE is represented as follows

(14) $$MSE=\frac{1}{N}\sum_{i=1}^{N}{\left(O_{i}-T_{j}\right)}^{2}$$

where$O_{i}$is the observed value at time, $T_{j}$is the target value at modelj; j = 1 to 64, and Nis the total number of observations per patient in our case, it is 122. This research uses all distance features of EEG data both for training and testing classifiers. The training was progressed regressively and the MSE Values of classifiers were decreased to minimum. At most all the classifiers are trained with zero training error of MSE.

The consolidated result analysis of correlation distance metric with the classifiers is shown in Table 4. The consolidated result analysis of city block distance metric with the classifiers is shown in Table 5. Table 6 explains the results of the cosine distance metric with the classifiers and Table 7 explains the results of the chebyshev distance metric with the classifiers. Table 8 explains the consolidated MSE and GDR result analysis with all the distance metrics and the classifiers.

**Table 4:** Consolidated Result Analysis of Correlation Distance metric with classifiers.

Classifiers	PI(%)	Sensitivity (%)	Specificity (%)	Accuracy (%)
Adaboost.RT	89.98	90.63	100	95.315
Ridge Based Modified Adaboost.RT dependent on Soft Thresholding	98.93	97.98	100	98.99
Lasso Based Modified Adaboost.RT dependent on Soft Thresholding	96.31292	96.4541	100	98.22705
Random Forest	95.65	95.83	100	97.915
RBF	97.87	97.92	100	98.96
MLP	81.47	84.38	100	92.19
Linear SVM	84.315	86.46	100	93.23
Polynomial SVM	82.93	85.42	100	92.71
RBF SVM	86.37	88.0225	100	94.01125
NBC	85.7	87.5	100	93.75
K-means	51.163	67.188	100	83.594
KNN	84.315	86.46	100	93.23

**Table 5:** Consolidated Result Analysis of City Block Distance metric with classifiers.

Classifiers	PI(%)	Sensitivity (%)	Specificity (%)	Accuracy (%)
Adaboost.RT	83.31458	86.77778	100	93.38889
Ridge Based Modified Adaboost.RT dependent on Soft Thresholding	97.87	97.92	100	98.96
Lasso Based Modified Adaboost.RT dependent on Soft Thresholding	97.41	97.48	100	98.74
Random Forest	85.7	87.5	100	93.75
RBF	90.78	91.15	100	95.575
MLP	94.49	94.79	100	97.395
Linear SVM	77.925	100	80.225	90.1125
Polynomial SVM	84.315	100	86.46	93.23
RBF SVM	89.98	90.63	100	95.315
NBC	78.465	100	82.295	91.1475
K-means	66.66	75	100	87.5
KNN	88.38	100	89.59	94.795

**Table 6:** Consolidated Result Analysis of Cosine Distance metric with classifiers.

Classifiers	PI(%)	Sensitivity (%)	Specificity (%)	Accuracy (%)
Adaboost.RT	76.92	81.25	100	90.625
Ridge Based Modified Adaboost.RT dependent on Soft Thresholding	94.49	94.79	100	97.395
Lasso Based Modified Adaboost.RT dependent on Soft Thresholding	97.83	100	97.88	98.94
Random Forest	80.01	83.34	100	91.67
RBF	93.615	93.75	100	96.875
MLP	82.565	85.16	100	92.58
Linear SVM	88.38	89.59	100	94.795
Polynomial SVM	93.33	93.75	100	96.875
RBF SVM	96.76	96.875	100	98.4375
NBC	63.76	73.43	100	86.71
K-means	40	62.5	100	81.25
KNN	77.925	80.225	100	90.1125

**Table 7:** Consolidated Result Analysis of Chebyshev Distance metric with classifiers.

Classifiers	PI(%)	Sensitivity (%)	Specificity (%)	Accuracy (%)
Adaboost.RT	93.33	100	93.75	96.875
Ridge Based Modified Adaboost.RT dependent on Soft Thresholding	92.455	92.71	100	96.355
Lasso Based Modified Adaboost.RT dependent on Soft Thresholding	97.57681	98.40806	99.27778	98.84292
Random Forest	89.98	90.63	100	95.315
RBF	90.78	91.15	100	95.575
MLP	91.79	91.93	100	95.965
Linear SVM	85.7	100	87.5	93.75
Polynomial SVM	81.47	100	84.38	92.19
RBF SVM	92.017	92.19	100	96.095
NBC	63.76	100	73.43	86.71
K-means	51.163	67.188	100	83.594
KNN	75.86	78.15	100	89.075

**Table 8:** Consolidated MSE and GDR Result Analysis with Distance metric and Classifiers.

Consolidated Analysis	Correlation Distance Metric		City Block Distance Metric		Chebyshev Distance Metric		Cosine Distance Metric	
Classifiers	MSE	GDR	MSE	GDR	MSE	GDR	MSE	GDR
Adaboost.RT	1.44E-06	90.63	8.267E-06	86.77778	4.9E-07	93.33333	1.296E-05	81.25
Ridge Based Modified Adaboost.RT dependent on Soft Thresholding	1E-08	98.02	4E-08	97.92	8.1E-07	92.71	3.6E-07	94.79474
Lasso Based Modified Adaboost.RT dependent on Soft Thresholding	1.873E-07	96.46086	2.5E-09	97.48	1.239E-07	97.6705	4E-08	97.83482
Random Forest	2.5E-07	95.83958	4E-06	87.5	1.44E-06	90.63	9E-06	83.34
RBF	4E-08	97.92	1.21E-06	91.15	1.21E-06	91.15	6.4E-07	93.75
MLP	7.84E-06	84.38	3.6E-07	94.79	8.1E-07	91.93	5.76E-06	85.16
Linear SVM	4.84E-06	86.46	1.44E-05	75.32727	4E-06	85.71429	2.25E-06	89.59
Polynomial SVM	6.76E-06	85.42	4.84E-06	84.33958	7.29E-06	81.4885	6.4E-07	93.75
RBF SVM	2.89E-06	88.0225	1.44E-06	90.63	6.4E-07	92.19	9E-08	96.875
NBC	4E-06	87.5	1.09E-05	78.48593	2.3E-05	63.82979	2.21E-05	73.45
K-means	4.1E-05	67.18263	2.03E-05	75	4.1E-05	67.188	8.65E-05	62.5
KNN	5.76E-06	85.94	2.25E-06	88.3804	1.68E-05	78.12	1.37E-05	80.21

### 4.2 Comparison of previous works

The result analysis with the standard previous works with respect to automated detection of alcohol risk levels have been compared and analyzed with our work in Table 9.

**Table 9:** Comparison of our works with previous works.

Authors	Features Obtained	Classification technique	Classification Accuracy (%)
Patidar et al [44]	Correlation Entropy	LS -SVM	97.02
Faust et al [45]	Higher Order Spectra (HOS) Features	Fuzzy Sugeno Classifier (FSC)	92.40
Acharya et al [18]	Entropy, HOS and LLE	SVM with different kernels	91.7
Kannathal et al [46]	CD and entropy	Distinct ranges	90
Proposed Work	CD with the Correlation Distance Metrics	Adaboost.RT	95.315
		Ridge Based Modified Adaboost.RT dependent on Soft Thresholding	98.99
		Lasso Based Modified Adaboost.RT dependent on Soft Thresholding	98.227
		Random Forest	97.915
		RBF	98.96
		MLP	92.19
		Linear SVM	93.23
		Polynomial SVM	92.71
		RBF SVM	94.011
		NBC	93.75
		K-means	83.594
		KNN	93.23
Proposed Work	CD with the City Block Distance Metrics	Adaboost.RT	93.388
		Ridge Based Modified Adaboost.RT dependent on Soft Thresholding	98.96
		Lasso Based Modified Adaboost.RT dependent on Soft Thresholding	98.74
		Random Forest	93.75
		RBF	95.575
		MLP	97.395
		Linear SVM	90.112
		Polynomial SVM	93.23
		RBF SVM	95.315
		NBC	91.147
		K-means	87.5
		KNN	94.795
Proposed Work	CD with the Cosine Distance Metrics	Adaboost.RT	90.625
		Ridge Based Modified Adaboost.RT dependent on Soft Thresholding	97.395
		Lasso Based Modified Adaboost.RT dependent on Soft Thresholding	98.94
		Random Forest	91.67
		RBF	96.875
		MLP	92.58
		Linear SVM	94.795
		Polynomial SVM	96.875
		RBF SVM	98.437
		NBC	86.71
		K-means	81.25
		KNN	90.1125
Proposed Work	CD with the Chebyshev Distance Metrics	Adaboost.RT	96.875
		Ridge Based Modified Adaboost.RT dependent on Soft Thresholding	96.355
		Lasso Based Modified Adaboost.RT dependent on Soft Thresholding	98.842
		Random Forest	95.315
		RBF	95.575
		MLP	95.965
		Linear SVM	93.75
		Polynomial SVM	92.19
		RBF SVM	96.095
		NBC	86.71
		K-means	83.594
		KNN	89.075

The main contribution in this work is the usage of CD for clustering and feature extraction and then using distance metrics for feature selection before classifying it using different post classifiers to determine the most efficient classification performance of alcoholism identification. In the last decade using a lot of linear and non-linear methods, many attempts have been utilized to discriminate alcoholic signals from normal signals. As depicted in Table 9, Patidar utilized a technique based on Tunable Q-wavelet transform (TQWT) and correntropy to identify the important differences in the alcoholic EEG signals and classify it with LS-SVM and reported a classification accuracy of 97.02% [44]. Faust et al utilized HOS features with FSC and obtained a classification accuracy of 92.40% [45]. Acharya et.al utilized approximate entropy, adaptive entropy, and HOS scheme to classify using LS-SVM and reported a classification accuracy of about 91.7% [18]. Kannathal et al. utilized CD and entropy to get the required features for alcohol detection from EEG signals and these features were helpful in the measurement of correlation and self-similarity properties and a classification accuracy of 90% was obtained [46].

In this study, about twelve different classifiers were utilized for the classification of alcoholism from EEG signals and the results show that when CD is utilized with correlation distance metrics and classified with Ridge based Modified Adaboost.RT with soft thresholding a good classification accuracy of 98.99% is obtained and when it is classified with Lasso based Modified Adaboost.RT with soft thresholding a good classification accuracy of 98.22% is obtained. When classifying with K means classifier, a low classification accuracy of 83.59% is obtained and while classifying with Random forest and RBF a classification accuracy of about 97.91% and 98.96% were reported respectively. Similarly, when CD is utilized with city block distance metrics and classified with Ridge based Modified Adaboost.RT with soft thresholding a high classification accuracy of 98.96% is obtained and when it is classified with Lasso based Modified Adaboost.RT with soft thresholding technique a high classification accuracy of 98.74% is obtained. With K-means classifier, it had a low classification accuracy of 87.5% and with MLP and RBF it reported an average classification accuracy of 97.39% and 95.57% respectively. Considering the analysis of CD with cosine distance metrics and classified with Ridge based Modified Adaboost.RT with soft thresholding technique a high classification accuracy of 97.39% is obtained and when it is classified with Lasso based Modified Adaboost.RT with soft thresholding technique a high classification accuracy of 98.94% is obtained. K-means and NBC produced a pretty less classification accuracy of about 81.25% and 86.71% respectively. With RBF and with SVM based on RBF kernel, it produced a classification accuracy of 96.87% and 98.43% respectively. When the CD is dealt with Chebyshev distance metrics for analysis and then when it is classified with Ridge based Modified Adaboost.RT with soft thresholding technique a high classification accuracy of 96.35% is obtained and when it is classified with Lasso based Modified Adaboost.RT with soft thresholding technique a good classification accuracy of 98.84% is obtained. With NBC and KNN, a less classification accuracy of about 86.71% and 89.07% is obtained and a comparatively good classification accuracy of 95.57%, 95.96% and 96.09% is obtained when utilizing RBF, MLP and SVM with RBF kernel.

The primary advantages are the efficiency and versatility of the system as it supports both robustness and reliability as 10-fold cross validation approach is proved to be more versatile yielding good results. Installation of this technique is quite easier and there is zero inter-observer variability. This proposed framework seems to be good in the area of alcoholic EEG signal classification, as prior to feature extraction basic pre-processing steps were taken and so the computation is fast and simple.

## 5 Conclusion and future work

This paper incorporates the uses of signal processing and pattern recognition techniques for alcoholic risk level detection using non-linear methods. In this work, CD was utilized for clustering and then using suitable distance metrics the features were extracted. Later the classification was done using two proposed classifiers and ten existing classifiers and the performance metrics was analyzed. It is concluded that using a methodology of clustering with CD and then feature extraction with distance metrics and classifying it with appropriate classifiers yields best results and follows a very systematic methodology. In this work, overall a very good classification accuracy of 98.99% was obtained when CD with correlation distance metrics for feature extraction was classified with Ridge based Modified Adaboost.RT classifier incorporating soft thresholding technique. A very good classification accuracy of 98.96% was obtained when CD with city block distance metrics for feature extraction was classified with Ridge based Modified Adaboost.RT classifier incorporating soft thresholding technique. A very good classification accuracy of 98.94% was obtained when CD with cosine distance block distance metrics for feature extraction was classified with Lasso based Modified Adaboost.RT classifier incorporating soft thresholding technique. A very good classification accuracy of 98.84% was obtained when CD with chebyshev distance metrics for feature extraction was classified with Lasso based Modified Adaboost.RT classifier incorporating soft thresholding technique. Therefore, the proposed methodology performs well with all the kinds of distance metrics and the classifiers utilized here yielding good classification accuracy. Future works aim to study and perform the analysis with other different type of features for the efficient alcohol risk level classification.

## Declarations

### Author contribution statement

S. K. Prabhakar, H. Rajaguru: Conceived and designed the experiments; Performed the experiments; Analyzed and interpreted the data; Contributed reagents, materials, analysis tools or data; Wrote the paper.

### Funding statement

This research did not receive any specific grant from funding agencies in the public, commercial, or not-for-profit sectors.

### Data availability statement

Data will be made available on request.

### Declaration of interests statement

The authors declare no conflict of interest.

### Additional information

No additional information is available for this paper.

## References

[1] Oscar-Berman M., Marinkovi K. (2007). Alcohol: effects on neurobehavioral functions and the brain. Neuropsychol. Rev..

[2] Das D., Zhou S., Lee J.D. (2012). Differentiating alcohol-induced driving behavior using steering wheel signals. IEEE Trans. Intell. Transport. Syst..

[3] Sripada C.S., Angstadt M., McNamara P., King A.C., Phan K.L. (2011). Effects of alcohol on brain responses to social signals of threat in humans. Neuroimage.

[4] Prabhakar S.K., Rajaguru H., Lee S.-W. (2019). A comprehensive analysis of alcoholic EEG signals with detrend fluctuation analysis and post classifiers. Seventh International Winter Conference on Brain-Computer Interface, 18–20 February 2019.

[5] Prabhakar S.K., Rajaguru H. (2017). Softmax discriminant classifier for detection of risk levels in alcoholic EEG signals. IEEE Proceedings of the International Conference on Computing Methodologies and Communication (ICCMC 2017).

[6] Prabhakar S.K., Rajaguru H. (October 19-20, 2017). Application of thresholding in correlation dimension for alcoholic risk level detection in EEG signals. 2nd IEEE International Conference on Communication and Electronics Systems.

[7] Prabhakar S.K., Rajaguru H. (2016). Code converters with city block distance measures for classifying epilepsy from EEG signals. Procedia Comput. Sci..

[8] Han S.-Y., Kwak N.-S., Oh T., Lee S.-W. (2020). Classification of pilots’ mental states using a multimodal deep learning network. Biocybern. Biomed. Eng..

[9] Jeong J.-H., Yu B.-W., Lee D.-H., Lee S.-W. (2019). Classification of drowsiness levels based on a deep spatio-temporal convolutional bidirectional LSTM network using Electroencephalography signals. Brain Sci..

[10] Harikumar R., Prabhakar S.K. (April 2015). fuzzy techniques and aggregation operators in classification of epilepsy risk levels for diabetic patients using EEG signals and cerebral blood flow. J. Biomater. Tissue Eng..

[11] Huang H., Liu X., Jin Y., Lee S.-W., Wee C.-Y., Shen D. (2019). Enhancing the representation of functional connectivity networks by fusing multi-view information for autism spectrum disorder diagnosis. Hum. Brain Mapp..

[12] Suk H.-I., Lee S.-W., Shen D. (2016). Deep sparse multi-task learning for feature selection in Alzheimer’s disease diagnosis. Brain Struct. Funct..

[13] Kim D.-H., Kim L., Park W., Chang W.-H., Kim Y.-H., Lee S.-W., Kwon G.-H. (2015). Analysis of time-dependent brain network on active and MI tasks for chronic stroke patients. PloS One.

[14] Lee M., Baird B., Gosseries O., Nieminen J.O., Boly M., Postle B., Tononi G., Lee S.-W. (2019). Connectivity differences between consciousness and unconsciousness in non-rapid eye movement sleep: a TMS–EEG study. Sci. Rep..

[15] Lee S.-B., Kim H.-J., Kim H., Jeong J.-H., Lee S.-W., Kim D.-J. (2019). Comparative analysis of features extracted from EEG spatial, spectral and temporal domains for binary and multiclass motor imagery classification. Inf. Sci..

[16] Lee M., Song C.-B., Shin G.-H., Lee S.-W. (2019). Possible effect of binaural beat combined with autonomous sensory meridian response for inducing sleep. Front. Hum. Neurosci..

[17] Prabhakar S.K., Rajaguru H., Lee S.-W. (2020). A framework for schizophrenia EEG signal classification with nature inspired optimization algorithms. IEEE Access.

[18] Acharya U.R., Sree S.V., Chattopadhyay S., S Suri J. (2012). Automated diagnosis of normal and alcoholic EEG signals. Int. J. Neural Syst..

[19] Zhu G., Li Y., Wen P. (2011). Evaluating functional connectivity in alcoholics based on maximal weight matching. J. Adv. Comput. Intell. Intell. Inf..

[20] Taran S., Bajaj V. (5 2018). Rhythm-based identification of alcohol EEG signals. IET Sci. Meas. Technol..

[21] Mumtaz W., Vuong P.L., Xia L., Malik A.S., Rashid R.B.A. (April 2017). An EEG-based machine learning method to screen alcohol use disorder. Cogn. Neurodyn..

[22] Sharma M., Deb D., Acharya U.R. (2018). A novel three-band orthogonal wavelet filter bank method for an automated identification of alcoholic EEG signals. Appl. Intell..

[23] Acharya U.R., Sree S.V., Swapna G G., Martis R.J., Suri J.S. (2013). Automated EEG analysis of epilepsy: a review. Knowl-Based Syst.

[24] Rajaguru H., Prabhakar S.K. (2018). A hybrid classification model using artificial bee colony with particle swarm optimization and minimum relative entropy as post classifier for epilepsy classification. Comput. Vis. Bioinspired Comput. Lect. Notes Comput. Vis. Biomech..

[25] Iasemidis L.D., Shiau D.S., Chaovalitwongse W., Sackellares J.C., Pardalos P.M., Príncipe J.C. (2003). Adaptive epileptic seizure prediction system. IEEE Trans. Biomed. Eng..

[26] Lehnertz K. (1999). Non-linear time series analysis of intracranial EEG recordings in patients with epilepsy – an overview. Int. J. Psychophysiol..

[27] Subasi A. (2005). Epileptic seizure detection using dynamic wavelet network. Expert Syst. Appl..

[28] UCI KDD database. https://archive.ics.uci.edu/ml/datasets/eegdatabase.

[29] Osorio I., Harrison M.A.F., Lai Y.-C., Frei M.G. (2001). Observations on the application of the correlation dimension and correlation integral to the prediction of seizures. J. Clin. Neurophysiol..

[30] Ocak H. (2009). Automatic detection of epileptic seizures in EEG using discrete wavelet transform and approximate entropy. Expert Syst. Appl..

[31] Li R., Zhong W., Zhu L. (2012). Feature screening via distance correlation learning. J. Am. Stat. Assoc..

[32] Melter R.A. (September 1987). Some characterizations of city block distance. Pattern Recogn. Lett..

[33] Borwein M. (2007). Proximity and Chebyshev sets. Opt. Lett..

[34] Li D., Cheng C. (2002). New similarity measures of intuitionistic fuzzy sets and application to pattern recognition. Pattern Recogn. Lett..

[35] Shrestha D.L., Solomatine D.P. (2006). Experiments with AdaBoost.RT, an improved boosting scheme for regression. Neural Comput..

[36] Piepho H.P. (2009). Ridge regression and extensions for genomewide selection in maize. Crop Sci..

[37] Algamal Z.Y., Lee M.H. (2014). Adjusted adaptive LASSO in high-dimensional Poisson regression model. Mod. Appl. Sci. (MAS).

[38] Nguyen C., Wang Y., Nguyen H.N. (2013). Random forest classifier combined with feature selection for breast cancer diagnosis and prognostic. J. Biomed. Sci. Eng..

[39] Chaudhuri B.B., Bhattacharya U. (2000). Efficient training and improved performance of multilayer perceptron in pattern classification. Neurocomputing.

[40] Mirowski P.W., LeCun Y., Madhavan D., Kuzniecky R. (2008). Comparing SVM and convolutional networks for epileptic seizure prediction from intracranial EEG. 2008 IEEE Workshop on Machine Learning for Signal Processing.

[41] Zavar M., Rahati S., Akbarzadeh-T M.R., Ghasemifard H. (2011). Evolutionary model selection in a wavelet-based support vector machine for automated seizure detection. Expert Syst. Appl..

[42] Faraoun K.M., Boukelif A. (2007). Neural networks learning improvement using the *K*-means clustering algorithm to detect network intrusions. Int. J. Comput. Intell..

[43] Altman (1992). An introduction to kernel and nearest-neighbor nonparametric regression. Am. Statistician.

[44] Patidar S., Pachori R.B., Upadhyay A., Acharya U.R. (2017). An integrated alcoholic index using tunable-Q wavelet transform based features extracted from EEG signals for diagnosis of alcoholism. Appl. Soft Comput..

[45] Faust O., Yanti R., Yu W. (2013). Automated detection of alcohol related changes in electroencephalograph signals. J. Med. Imaging. Health Inform..

[46] Kannathal N., Acharya U.R., Lim C.M., Sadasivan P.K. (2005). Characterization of EEG-A comparative study Comput. Methods Progr. Biomed..

